# Prevalence and related factors of irritable bowel syndrome in women with chronic pelvic pain

**DOI:** 10.1016/j.clinsp.2026.100884

**Published:** 2026-02-26

**Authors:** João Nogueira Neto, Kamilly Ieda Silva Veigas, Lyvia Maria Rodrigues de Sousa Gomes, Leonardo Carvalho Silva, Júlio Cesar Rosa e Silva, Plínio da Cunha Leal

**Affiliations:** a Department of Medicine, Universidade Federal do Maranhão, São Luís, MA, Brazil; b Department of Gynecology and Obstetrics, Universidade de São Paulo, Ribeirão Preto, SP, Brazil

**Keywords:** Chronic pain, Pelvic pain, Irritable bowel syndrome

## Abstract

**Purpose:**

To evaluate the prevalence of Irritable Bowel Syndrome (IBS) and its related sociodemographic, nociceptive, neuropathic, nociplastic, psychosocial, and systemic factors in patients with Chronic Pelvic Pain (CPP).

**Materials and methods:**

This observational, prospective, and analytical study was conducted at a Pelvic Pain Outpatient Clinic between August 2021 and March 2022 with patients diagnosed with CPP. The following instruments were applied: IBS was diagnosed using the Rome IV criteria, the Questionnaire for Chronic Pelvic Pain Assessment (QCPPA) of the International Pelvic Pain Society (IPPS), the Depression, Anxiety and Stress Scale (DASS-21), the Douleur Neuropathique 4 questionnaire (DN4), and the Central Sensitization Inventory (CSI).

**Results:**

In this study, 111 women with CPP were evaluated. The prevalence of IBS was 53.15%. Patients with IBS showed significantly higher frequencies of myofascial pain (p < 0.001), neuropathic pain (p = 0.026), and central sensitization (p = 0.010). Binary logistic regression identified myofascial pain (OR = 7.614; 95% CI: 2.12–22.88; p = 0.001) as an independent predictor of IBS, while infertility showed a protective association with IBS, with an adjusted odds ratio of 0.209 (95% CI: 0.048–0.915; p = 0.038).

**Conclusion:**

Women with CPP have a high prevalence of IBS. In addition to the association with classic factors, this study also observed a significant association with neuropathic pain and pain centralization. A significant association with myofascial syndrome was observed, which appeared as a strong predictor of IBS in the adjusted model.

## Highlights

• Study found 53.15% prevalence of IBS in women with Chronic Pelvic Pain (CPP).
• No significant differences in sociodemographic factors between IBS and non-IBS patients.
• IBS patients had higher rates of myofascial pain, neuropathic pain, and sensitization.
• Multiple assessment tools were used, including the Rome IV criteria for IBS diagnosis.
• Results highlight the link between IBS and chronic pelvic pain, necessitating better care.

## Introduction

Chronic pain is a complex and multifactorial condition with nociceptive, neuropathic and nociplastic causes.^1^ The American College of Obstetricians and Gynecologists (ACOG) and the International Federation of Gynecology and Obstetrics (FIGO) define Chronic Pelvic Pain (CPP) as pain symptoms perceived to originate from pelvic organs/structures, typically lasting more than 3 to 6-months.^1^^,^^2^ It is often associated with negative cognitive, behavioral, sexual, and emotional consequences as well as with symptoms indicating dysfunction of the lower urinary tract, sexual, bowel, pelvic floor, myofascial, or gynecological organs.^1^^,^^2^

FIGO and the International Pelvic Pain Society (IPPS) have classified CPP into the acronym letters “R U MOVVING SOMe”, which represents: Reproductive, Urinary, Musculoskeletal, Other (not otherwise classified), Vulvovaginal, Vascular, Idiopathic (no pain contributor identified), Neurologic, Gastrointestinal, Sensitization/Nociplastic, Overlapping pain conditions, and Mental health.^1^ Gastrointestinal causes describe conditions related to the digestive system that can manifest as CPP, often associated with bloating and bowel dysfunction. The most common condition included in this category is Irritable Bowel Syndrome (IBS).^1^^,^^2^

IBS is characterized by altered bidirectional communication between the gut and the CNS, specifically the brain. Notably, IBS is part of a group of functional gastrointestinal disorders called Disorders of Gut­Brain Interaction (DGBI).^1^ These conditions are characterized by altered bidirectional communication between the gut and the CNS, specifically the brain. In DGBI, disturbances in this interaction lead to gastrointestinal symptoms, including abdominal pain, bloating, and altered bowel habits, without identifiable structural or biochemical abnormalities in the gut. The underlying pathophysiology is multifactorial, encompassing altered gastrointestinal motility, visceral hypersensitivity, alterations in mucosal and immune function of the gut, changes in microbiota, CNS processing abnormalities, and psychological factors.^1^

The prevalence of IBS in females with CPP ranges from 5.7% to 85.4%.^3^, ^4^, ^5^, ^6^, ^7^ IBS is diagnosed based on the ROME criteria, which consider the presence of abdominal pain associated with changes in defecation frequency and stool consistency, provided there are no alarm signs such as rectal bleeding, weight loss, or other findings suggestive of alternative diagnoses.^8^

The Rome IV criteria are used to screen for IBS. A positive diagnosis requires recurrent abdominal pain occurring at least once per week, associated with two or more of the following: defecation-related pain (≥ 30%), changes in stool frequency (≥30%), or changes in stool form (≥ 30%), with symptom onset at least six months prior to diagnosis. The criteria also enable classification into subtypes: constipation-predominant, diarrhea-predominant, mixed, or unclassified. Rome IV has a sensitivity of 75%, specificity of 80%, and a negative predictive value of 91%.^9^

Age, cystitis, endometriosis, adenomyosis, anxiety, depression, dyspareunia, pain duration, low back pain, levels of somatization, inflammatory markers, intestinal dysbiosis, fibromyalgia, migraine, and a history of physical or sexual abuse are factors associated with IBS and CPP.^3^, ^4^, ^5^, ^6^, ^7^

Due to the scarcity of studies evaluating CPP with or without IBS, in addition to a better understanding of the pathophysiology of CPP and IBS, with the emergence of validated forms to evaluate nociceptive, neurological, and neuroplastic changes, new studies are necessary for better assistance, diagnosis, adequate treatment, and consequent improvement in the quality of life of patients with CPP.

This study aimed to evaluate the prevalence of IBS and the associated sociodemographic, nociceptive, neuropathic, nociplastic, psychosocial, and systemic factors in women with CPP.

## Materials and methods

### Study design and setting

This observational, prospective, and analytical study was conducted at a high-complexity hospital's Pelvic Pain Outpatient Clinic, in São Luís, Maranhão, Brazil, between August 2021 and March 2022.

### Participants

The sample size calculation was performed using GPower software version 3.1. An analysis of two independent proportions was performed, assuming a significance level of 5% (α = 0.05) and statistical power of 80% (1−β = 0.80), based on the expected proportion of IBS of 38% in individuals with chronic pelvic pain and 62% without pain. A sample of 106 women was determined, adding 10% for the chance of loss, resulting in the final expected sample of 116 patients.

Women diagnosed with chronic pelvic pain according to the American College of Obstetricians and Gynecologists (ACOG)^2^ were eligible, with the inclusion criteria being: age 18-years or older, who agreed to participate in the study and signed the Informed Consent Form, and who were physically and mentally able to understand the objectives of the study. Participants who were already undergoing treatment for CPP in other services were excluded.

### Data collection and instruments

During the outpatient consultation, patients diagnosed with CPP who agreed to participate in the study and who had not started treatment received five printed questionnaires, in addition to the Informed Consent Form.

The Rome IV criteria were used to diagnose IBS, including the presence of abdominal pain, on average, at least one day a week in the last three months, accompanied by changes in the form, frequency of stools, and pain relief after evacuation.^10^ All selected patients were referred to gastroenterology outpatient clinics, in which they underwent fecal occult blood tests, colonoscopy, or other necessary tests to rule out the presence of polyps or intestinal tumors with the possibility of cancer, inflammatory bowel disease, or any other structural change.

The validated and translated version in Portuguese of the Questionnaire for Chronic Pelvic Pain Assessment (QCPPA) from the International Pelvic Pain Society (IPPS) was applied.^11^ For this study, data were extracted regarding age, marital status, level of education, obstetric history, duration of pain, pain assessment using the McGill scale, presence or absence of common pelvic symptoms or pathologies in CPP such as dysmenorrhea, dyspareunia, endometriosis, adenomyosis, leiomyomatosis, as well as migraine, high blood pressure, diabetes, pelvic inflammatory disease, fibromyalgia, physical or sexual abuse, painful bladder syndrome, pelvic congestion syndrome, infertility, vulvodynia or perianal pain.

For the analysis of Pelvic Pain and Urgency/Frequency (PUF), also included in the QCPPA/IPPS, urinary symptoms were based on research by Parsons et al.,^12^ and the result was considered positive when the score was greater than or equal to 15, according to Sammarco et al.^13^

The validated short version of the Depression, Anxiety, and Stress Scale (DASS-21) was also administered.^14^ This instrument measures three dimensions: depression, anxiety, and stress. It comprises three subscales: Depression (items 3, 5, 10, 13, 16, 17, 21), Anxiety (items 2, 4, 7, 9, 15, 19, 20), and Stress (items 1, 6, 8, 11, 12, 14, 18). Each item has four response options ranging from 0 (does not apply to me) to 3 (applies to me most of the time). Scores for each subscale are summed and multiplied by two, yielding a range from 0 to 42 per subscale. The classification of stress scores is: 0‒14 = normal, 15‒18 = mild; 19‒25 moderate, 26‒33 = severe and 34‒42 = extremely severe. For anxiety, it is 0‒7 normal, 8‒9 = mild, 10‒14 = moderate, 15‒19 = severe and 20‒42 extremely severe. For depression: 0‒9 = normal; 10‒13 = mild; 14‒20 = moderate; 21‒27 = severe and 28‒42 = extremely severe.

The presence or absence of myofascial pelvic pain was evaluated by musculoskeletal sensitive points in women with CPP, including the pelvic floor, abdomen, groin, and inner thigh, as described by Sanses et al.^15^

Neuropathic pain characteristics were assessed with the Douleur Neuropathique-4 (DN4) questionnaire,^16^ which is simple to administer and effectively supports the diagnosis of neuropathic pain. The first seven assess pain qualities (burning, painful cold, electric shocks) and accompanying abnormal sensations (tingling, pins and needles, numbness, itching). The remaining three items are based on neurological examination findings in the painful area (hypoesthesia to touch, hypoesthesia to pinprick, tactile allodynia). Each positive item scores 1, and the total score ranges from 0 to 10. A score of 4 or higher indicates neuropathic pain.

The Central Sensitization Inventory (CSI), a self-administered questionnaire, was used to assess pain centralization and related symptoms. Responses are rated on a 5-point Likert scale: Never (0), Rarely (1), Sometimes (2), Often (3), and Always (4), yielding a total score from 0 to 100. CSI was classified as subclinical (score 0 to 29), mild (30 to 39), moderate (40 to 49), severe (50 to 59), and extreme (60 to 100), according to Neblett et al.^17^

### Ethical aspects

The study was conducted following the Declaration of Helsinki, approved by the Ethics Committee of the Hospital São Domingos, under CAAE: 43.223.721.00000.5085 and Approval Number: 6.967.258. The Free and Informed Consent was obtained from all participants.

### Data analysis

Data were tabulated in Microsoft Office Excel® (version 2019) (Redmond, WA, USA) and analyzed in SPSS (version 26) (Chicago, IL, USA). Categorical variables were expressed as absolute (n) and relative (%) frequencies, while numerical variables were reported as mean ± standard deviation or median (range: minimum to maximum). The Shapiro-Wilk test was used to assess normality. Missing data in this study were treated by excluding cases with incomplete information for each variable analyzed (listwise deletion).

Categorical variables were compared between patients with and without IBS using the Chi-square or Fisher's exact test. Based on data distribution, numerical variables were compared using the Student's *t*-test or the Mann-Whitney test.

Hierarchical binary logistic regression was performed to identify independent factors associated with IBS in women with CPP, divided into blocks: 1) Sociodemographic characteristics and gynecological history, 2) Comorbidities and gynecological health history, and 3) Scale of pain centralization, depression, anxiety, and stress. The model underwent Backwards Stepwise (Wald) selection, and only variables that remained statistically significant in the adjusted model were retained. Statistical significance was set at p < 0.05.

## Results

**Fig. 1:**
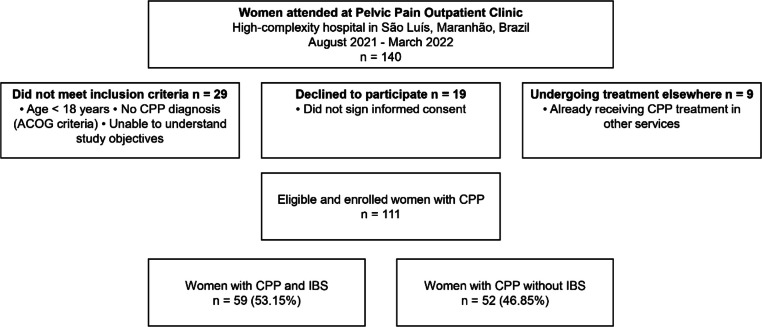
Inclusion and exclusion flowchart.

**Fig. 2:**
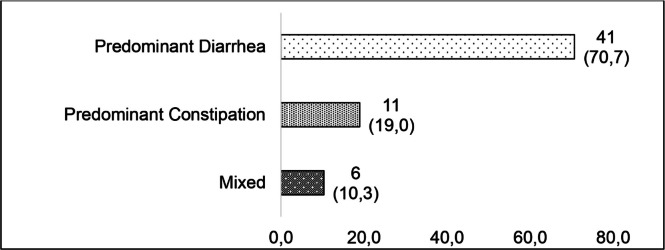
IBS subtypes of patients with chronic pelvic pain.

Of the 140 patients treated during the study period, 29 did not meet the inclusion criteria, 19 did not agree to participate, 9 were already undergoing treatment in other services, 111 women met the inclusion criteria, and 59 (53.15%) had IBS (Fig. 1). Among patients with IBS, predominance of diarrhea (71.7%) was observed in this population (Fig. 2).

No significant differences between patients with and without IBS regarding sociodemographic variables and gynecological history were observed. The mean age was similar between the groups (33.1 ± 7.6 vs. 31.9 ± 7.6 years), most participants were married (61.0% in the patients with IBS and 63.5% in those without IBS), and had completed higher education in both groups (52.5% and 42.3%, respectively). The majority of patients had never been pregnant (49.2% with IBS and 48.1% without IBS), nor had they experienced deliveries (61.0% and 53.8%, respectively) or abortions (83.1% and 80.8%), and the absence of ectopic pregnancy was predominant in both groups (96.6% with IBS and 100% without IBS). The median duration of pain was longer in patients with IBS (33-months) compared to those without IBS (24-months), although this difference was not statistically significant (p = 0.775) (Table 1).

**Table 1:** Sociodemographic characteristics and gynecological history of women with and without irritable bowel syndrome. São Luís, Maranhão, 2025.

Variables	With IBS	Without IBS	p-value¥
	n (%)	n (%)	
Age (years), Md ± SD	33.1 ± 7.6	31.9 ± 7.6	0.989^a^
Marital status			
Single	23 (39.0)	17 (32.7)	0.323^b^
Married	36 (61.0)	33 (63.5)	
Divorced	0 (0.0)	2 (3.8)	
Education			
Incomplete high school	0 (0.0)	1 (1.9)	0.548^b^
Completed high school	16 (27.1)	18 (34.6)	
Completed higher Education	31 (52.5)	22 (42.3)	
Postgraduate	12 (20.3)	11 (21.2)	
Number of pregnancies			
None	29 (49.2)	25 (48.1)	0.170
1 to 2	27 (45.8)	19 (36.5)	
3 to 4	3 (5.1)	8 (15.4)	
Parity			
None	36 (61.0)	28 (53.8)	0.097^b^
1 to 2	22 (37.3)	18 (34.6)	
3 to 4	1 (1.7)	6 (11.5)	
N° of abortions			
None	49 (83.1)	42 (80.8)	0.755
1 to 2	10 (16.9)	10 (19.2)	
Ectopic pregnancy			
Yes	2 (3.4)	0 (0.0)	0.280^b^
No	57 (96.6)	52 (100)	
Pain duration (months), Med (Min‒Max)	33 (6‒276)	24 (6‒240	0.775^c^
McGill, Med (Min‒Max)	18 (2‒42)	12.5 (1‒39)	0.071

IBS, Irritable Bowel Syndrome; n (%), Calculated based on the total number of participants in each group (column);

¥ Chi-Square;

a Student's *t*-test;

b Fisher's exact test;

c Mann-Whitney.

Most of the dependent variables showed a similar distribution between IBS and non-IBS patients, with no statistically significant differences, except for neuropathic pain and myofascial pain. A history of sexual abuse was reported by 44.1% of women with IBS and by 48.1% of those without IBS. The prevalence of systemic arterial hypertension was comparable between the groups (10.2% vs. 5.8%), as were rates of diabetes mellitus (3.4% vs. 1.9%), migraine (88.1% vs. 84.6%), fibromyalgia (13.6% vs. 9.6%) and infertility (16.9% vs. 19.2%). Dysmenorrhea was present in nearly all participants (94.9% vs. 100%), followed by dyspareunia (83.1% vs. 73.1%), endometriosis (69.5% vs. 63.5%), adenomyosis (47.5% vs. 42.3%), leiomyoma (27.1% vs. 19.2%), pelvic inflammatory disease (23.7% vs. 21.2%), pelvic congestion (66.1% vs. 57.7%), vulvodynia (47.5% vs. 38.5%), and perianal pain (20.3% vs. 13.5%). Myofascial pain was the most prevalent condition among participants with IBS (84.7%), while its prevalence was significantly lower in those without IBS (51.9%) (p < 0.001). Similarly, neuropathic pain was more frequent in IBS (47.5%) compared to those without IBS (26.9%) (p = 0.026) (Table 2).

**Table 2:** Comorbidities and gynecological health history of women with and without irritable bowel syndrome. São Luís, Maranhão, 2025.

Variables	With IBS	Without IBS	p-value¥
	n (%)	n (%)	
History of sexual abuse	26 (44.1)	25 (48.1)	0.672
Systemic arterial hypertension	6 (10.2)	3 (5.8)	0.312^a^
Diabetes mellitus	2 (3.4)	1 (1.9)	0.548^a^
Migraine	52 (88.1)	44 (84.6)	0.588
Fibromyalgia	8 (13.6)	5 (9.6)	0.519
Infertility	10 (16.9)	10 (19.2)	0.755
Dysmenorrhea	56 (94.9)	52 (100.0)	0.147^a^
Dyspareunia	49 (83.1)	38 (73.1)	0.203
Endometriosis	41 (69.5)	33 (63.5)	0.501
Adenomyosis	28 (47.5)	22 (42.3)	0.586
Leiomyoma	16 (27.1)	10 (19.2)	0.328
Pelvic inflammatory disease	14 (23.7)	11 (21.2)	0.746
Pelvic congestion	39 (66.1)	30 (57.7)	0.362
Vulvodynia	28 (47.5)	20 (38.5)	0.340
Perianal pain	12 (20.3)	7 (13.5)	0.337
Neuropathic pain	28 (47.5)	14 (26.9)	***0.026***
Myofascial pain	50 (84.7)	27 (51.9)	***<0.001***

IBS, Irritable Bowel Syndrome; n (%), Calculated based on the total number of participants in each group (column);

¥ Chi-Square;

a Fisher's Exact.

A significant difference was observed between the groups regarding pain centralization (p = 0.010), with a higher proportion of women with IBS classified as severe (32.4%) and extreme (38.2%) compared to those without IBS (19.5% and 24.4%, respectively). No significant differences were identified between the groups in the dimensions assessed by the DASS-21. In the depression subscale, most participants scored in the normal range in both with IBS (45.8%) and without IBS (38.5%), followed by those in the extremely severe category (20.3% vs. 23.1%). In the anxiety subscale, extremely severe levels were also the most frequent in both groups (33.9% vs. 38.5%). In the stress subscale, normal levels were most common (40.7% with IBS and 34.6% without IBS). Regarding pain intensity measured by the McGill scale, patients with IBS had a median score of 18 (2–42), compared to 12.5 (1–39) for those without IBS. Based on the PUF score, 54.2% of patients with IBS were classified as symptomatic, compared to 38.5% of those without IBS, although this difference was not statistically significant (p = 0.096) (Table 3).

**Table 3:** Scale of pain centralization, depression, anxiety, stress and pelvic pain symptoms of women with and without irritable bowel syndrome. São Luís, Maranhão, 2025.

Variables	With IBS	Without IBS	p-value¥
	n (%)	n (%)	
Pain centralization			
Subclinical	1 (2.9)	9 (22.0)	***0.010***^a^
Mild	5 (14.7)	6 (14.6)	
Moderate	4 (11.8)	8 (19.5)	
Severe	11 (32.4)	8 (19.5)	
Extreme	13 (38.2)	10 (24.4)	
DASS-21			
Depression			
Normal	27 (45.8)	20 (38.5)	0.767
Mild	8 (13.6)	5 (9.6)	
Moderate	7 (11.9)	10 (19.2)	
Severe	5 (8.5)	5 (9.6)	
Extremely severe	12 (20.3)	12 (23.1)	
Anxiety			
Normal	18 (30.5)	17 (32.7)	0.685
Mild	7 (11.9)	5 (9.6)	
Moderate	6 (10.2)	7 (13.5)	
Severe	8 (13.6)	3 (5.8)	
Extremely severe	20 (33.9)	20 (38.5)	
Stress			
Normal	24 (40.7)	18 (34.6)	0.492
Mild	7 (11.9)	7 (13.5)	
Moderate	9 (15.3)	8 (15.4)	
Severe	9 (15.3)	4 (7.7)	
Extremely severe	10 (16.9)	15 (28.8)	
PUF			
Positive	32 (54.2)	20 (38.5)	0.096 b
Negative	27 (45.8)	32 (61.5)	

IBS, Bowel Syndrome Irritable; DASS, Depression Anxiety and Stress Scale; PUF, Pelvic Pain and Urgency/Frequency; n (%): Calculated based on the total number of participants in each group (column);

¥ Chi-Square;

a Fisher's exact; ^b^Mann-Whitney.

Myofascial pain emerged as a strong independent predictor of IBS in women with CPP, with an adjusted odds ratio of 7.614 (95% CI: 2.12–22.88; p = 0.001). Women with myofascial pain had approximately 7.6-times higher odds of having IBS compared to those without myofascial pain. Infertility showed a protective association with IBS, with an adjusted odds ratio of 0.209 (95% CI: 0.048–0.915; p = 0.038). Women with infertility had approximately 79% lower odds of having IBS compared to those without infertility. The other variables did not present statistical relevance in the final model (Table 4).

**Table 4:** Binary logistic regression analysis of factors associated with irritable bowel syndrome in women with chronic pelvic pain. São Luís, Maranhão, 2025.

Variables	β	S.E.	Exp(B)	95% CI	p-value
Myofascial pain	2.030	0.599	7.614	2.12 ‒ 22.88	***0.001***
Infertility	-1.567	0.754	0.209	0.048 ‒ 0.915	***0.038***
Constant	-1.738	2.009	0.176		

Adjusted for: Marital status, Level of education, Number of pregnancies, Parity, Abortion; Systemic arterial hypertension, Diabetes mellitus, Dyspareunia, Endometriosis, Adenomyosis, Leiomyoma, Migraine, Neuropathic pain, Myofascial pain, Fibromyalgia, Pelvic inflammatory disease, Infertility, Pain centralization, Pelvic congestion, Vulvodynia, Perianal pain, History of sexual abuse, Depression, Anxiety, Stress, Pelvic Pain and Urgency/Frequency; β, regression coefficient; S.E., Standard Error; Exp(B), Odds Ratio; 95% CI, 95% Confidence Interval.

## Discussion

Pacientes com DPC com ou sem SII apresentaram características demográficas e nociceptivas semelhantes e, aparentemente, foi observada maior prevalência de alterações de dor neuropática, nociplástica e mista, bem como presença de dor miofascial.

The gut-brain axis is a bidirectional communication pathway connecting the gastrointestinal tract and the brain.^18^ The DGBI manifests as highly prevalent gastrointestinal disorders such as IBS, reflecting changes in the production of the autonomic nervous system and emotional changes, in addition to the involvement of disorders in the Hypothalamic-Pituitary-Adrenal (HPA) axis, inflammatory, immunological, microbiota, and serotonin alterations.^18^ Pharmacological therapies targeting DGBI in IBS have focused on the HPA axis, the microbiota, and the serotonergic system, and non-pharmacological holistic therapies include diet, neurostimulation, cognitive behavioral therapy, and hypnosis.^19^

In the present study, the authors found that the majority of patients with CPP also had IBS. This finding aligns with the literature, in which the reported prevalence of IBS in this population ranges from 5.7% to 85.4%.^3^, ^4^, ^5^, ^6^, ^7^ Sociodemographic characteristics did not differ significantly between groups, consistent with results from other recent studies.^3^, ^4^, ^5^, ^6^ Pain duration and intensity were two additional variables that showed no differences between groups. Although Lessa et al.^4^ reported that CPP lasting more than 12-months was more frequently associated with IBS, these findings did not reveal a significant association. Similarly, pain intensity was comparable between groups, as observed in the study by Tachawiwat and Cheewadhanaraks.^3^

The authors observed that symptoms such as dysmenorrhea and dyspareunia were quite frequent among both those with and without IBS, with no statistical difference, findings consistent with another study conducted in patients with CPP.^4^ A meta-analysis examining the relationship between primary dysmenorrhea and CPP, which included 32 studies, concluded that dysmenorrhea was generally associated with a 2.50-fold increase in the likelihood of chronic pain.^20^ Visceral etiologies such as endometriosis, adenomyosis, leiomyomatosis and interstitial cystitis did not show significant differences between groups, in agreement with the study by Tachawiwat and Cheewadhanaraks.^3^ However, the authors did not identify any studies specifically examining leiomyomatosis and the association with CPP and IBS.

Pelvic inflammatory disease, pelvic congestion syndrome, and fibromyalgia were more prevalent in the group with CPP and IBS, although without a significant difference.

Migraine was highly prevalent in both groups in the present study. Karp et al.^21^ reported the presence of migraine in 67% of patients with CPP with or without endometriosis in both groups, showing no significant difference between groups, findings that align with ours. Similarly, Lessa et al.^4^ found comparable results when analyzing CPP patients with or without IBS. In contrast, Choung et al.^5^ reported a higher prevalence of migraines in patients with CPP and IBS compared to those with IBS alone, and Georgescu et al.^6^ also found a significantly greater number of migraine cases in patients with CPP and IBS.

Systemic conditions such as arterial hypertension and diabetes were not related to the presence of IBS in patients with CPP in the present sample. However, Liao et al.,^22^ in a study investigating the association between prior IBS and chronic prostatitis/chronic pelvic pain syndrome, found evidence that these patients are significantly affected by previous IBS. They also reported that affected individuals were more likely than controls to have comorbid hypertension and coronary artery disease. Regarding diabetes, the authors found no studies in the literature examining its association with CPP, with or without IBS.

A recent study conducted by the present research group demonstrated that symptoms of depression, anxiety, and stress are common among women with CPP and that pain intensity is significantly associated with the presence of stress and anxiety but not with depression.^23^ In the present study, using the DASS-21 scale, the authors found no significant differences between the CPP groups with and without IBS. These findings are consistent with those of Lessa et al.,^4^ but contrast with those of Choung et al.,^5^ who reported a higher prevalence of depression in patients with IBS. Neither of those studies evaluated anxiety and/or stress. Although no difference was found between the groups in this study, its clinical relevance should be emphasized and researched, as these mental states are associated with other signs and symptoms of patients with CPP, leading to a loss of quality of life, and should be included in the treatment of these patients.

Studies have shown that sexual abuse is a significant risk factor for the worsening of CPP, possibly due to its association with various psychological and psychiatric comorbidities, such as anxiety, depression, and post-traumatic stress disorder. However, there is no clear consensus on the nature of this relationship. Additionally, the variability in screening methods for sexual abuse among women with CPP complicates comparisons across studies and hinders the development of consistent clinical guidelines.^24^, ^25^, ^26^, ^27^ In this sample, a history of physical and sexual abuse was reported in nearly half of the CPP cases, both with and without IBS. This finding contrasts with the results of Lessa et al.,^4^ who found that patients with CPP and IBS had significantly higher rates of abuse.

Vulvodynia was more frequent in patients with CPP and IBS than in those without IBS, although the difference was not statistically significant. Similarly, perianal pain, drug allergies, and infertility did not differ significantly between the groups.

In contrast, myofascial pain was significantly more prevalent in patients with CPP and IBS, as observed in the adjusted model, a finding consistent with the results of Georgescu et al.,^6^ who also reported a higher frequency of this condition in this population. These findings underscore the relevance of pelvic physiotherapy as a complementary treatment modality for patients with CPP, particularly those with IBS.

The inverse association between infertility and IBS suggests that women with infertility may represent a distinct clinical phenotype within the CPP population, characterized by different hormonal profiles, inflammatory markers, or gut microbiome compositions.

In the present study, patients with CPP and IBS exhibited significantly more neuropathic pain than those without IBS. Remarkably, recent studies involving similar patient populations have not evaluated this type of pain.^3^, ^4^, ^5^, ^6^ Regarding pain centralization, it was observed that patients with CPP and IBS had significantly higher levels of central sensitization compared to those without IBS, particularly in the severe and extreme categories.^28^, ^29^, ^30^ Although the authors did not find a specific explanation in the literature as to why patients with CPP and IBS might exhibit greater central sensitization than those with other visceral pain syndromes, the numerical trends in the data suggest that studies with larger sample sizes may yield more definitive conclusions.

Neuropathic pain, central sensitization, and psychological variables (depression, anxiety, stress), despite showing significance in bivariate analyses, did not remain as independent predictors in the final model. This suggests that their associations with IBS may be mediated through other pathways in the model.

Limitations of this study include the exclusion of patients undergoing treatment for chronic pelvic pain in other services, which may introduce selection bias. Its execution in a private hospital setting and the time required for patients to complete self-report instruments. Additionally, there is a potential risk that these self-reports may not accurately reflect the proper diagnosis or severity of the conditions assessed. This criterion was adopted to avoid interference from concomitant treatments, but it may restrict sample representativeness. Despite being an international standard, the Rome IV criteria carry a risk of misclassification of up to 25%. The cross-sectional design makes it impossible to infer causal relationships between the variables studied, so the results should be interpreted as associations rather than cause-and-effect relationships. Similarly, a larger sample could reveal even more clinically relevant differences. The absence of biological markers (inflammatory, hormonal, microbiome data) is also an important limitation. These limitations should be considered when interpreting the findings and considering their generalizability to other populations.

Multicenter studies are needed to expand the external validity of the findings, with larger sample sizes and prospective designs, comparative studies with different disease durations and treatments, and incorporating structured clinical interviews to validate diagnoses. The authors suggest that future investigations explore inflammatory markers, gut-brain axis dysfunction, and other biological mechanisms.

## Conclusion

Women with CPP have a high prevalence of IBS. In addition to the relation with classic factors such as age, endometriosis, adenomyosis, cystitis, dyspareunia, and pain duration, this research also found myofascial syndrome, neuropathic pain, and pain centralization. Regression modeling revealed that myofascial pain is the strongest independent predictor of IBS, maintaining its association even after adjustment for sociodemographic, gynecological, and psychological factors.

## Abbreviations

American College of Obstetricians and Gynecologists (ACOG); Central Sensitization Inventory (CSI); Chronic pelvic pain (CPP); Depression, Anxiety and Stress Scale (DASS-21); Douleur Neuropathique 4 questionnaire (DN4); International Federation of Gynecology and Obstetrics (FIGO); Gastrointestinal (GI); International Pelvic Pain Society (IPPS); Irritable Bowel Syndrome (IBS); Questionnaire for Chronic Pelvic Pain Assessment (QCPPA).

## Funding

The authors did not receive support from any organization for the submitted work.

## Data availability statement

The datasets generated and/or analyzed during the current study are available from the corresponding author upon reasonable request.

## CRediT authorship contribution statement

**João Nogueira Neto:** Conceptualization, Data curation, Formal analysis, Investigation, Methodology, Writing – original draft. **Kamilly Ieda Silva Veigas:** Data curation, Formal analysis, Investigation. **Lyvia Maria Rodrigues de Sousa Gomes:** Supervision, Validation, Visualization. **Leonardo Carvalho Silva:** Supervision, Validation, Visualization. **Júlio Cesar Rosa e Silva:** Visualization, Writing – review & editing. **Plínio da Cunha Leal:** Supervision, Validation, Visualization, Writing – review & editing.

## Declaration of competing interest

The authors declare that they have no known competing financial interests or personal relationships that could have appeared to influence the work reported in this paper.
